# Supplementary material for: Microbial stimulation of oxytocin release from the intestinal epithelium via secretin signaling
Source: Gut Microbes. 2023 Sep 12;15(2):2256043. doi: 10.1080/19490976.2023.2256043 (PMC10498800; doi:10.1080/19490976.2023.2256043)
Supplement: Supplemental Material [file KGMI_A_2256043_SM3541.docx]

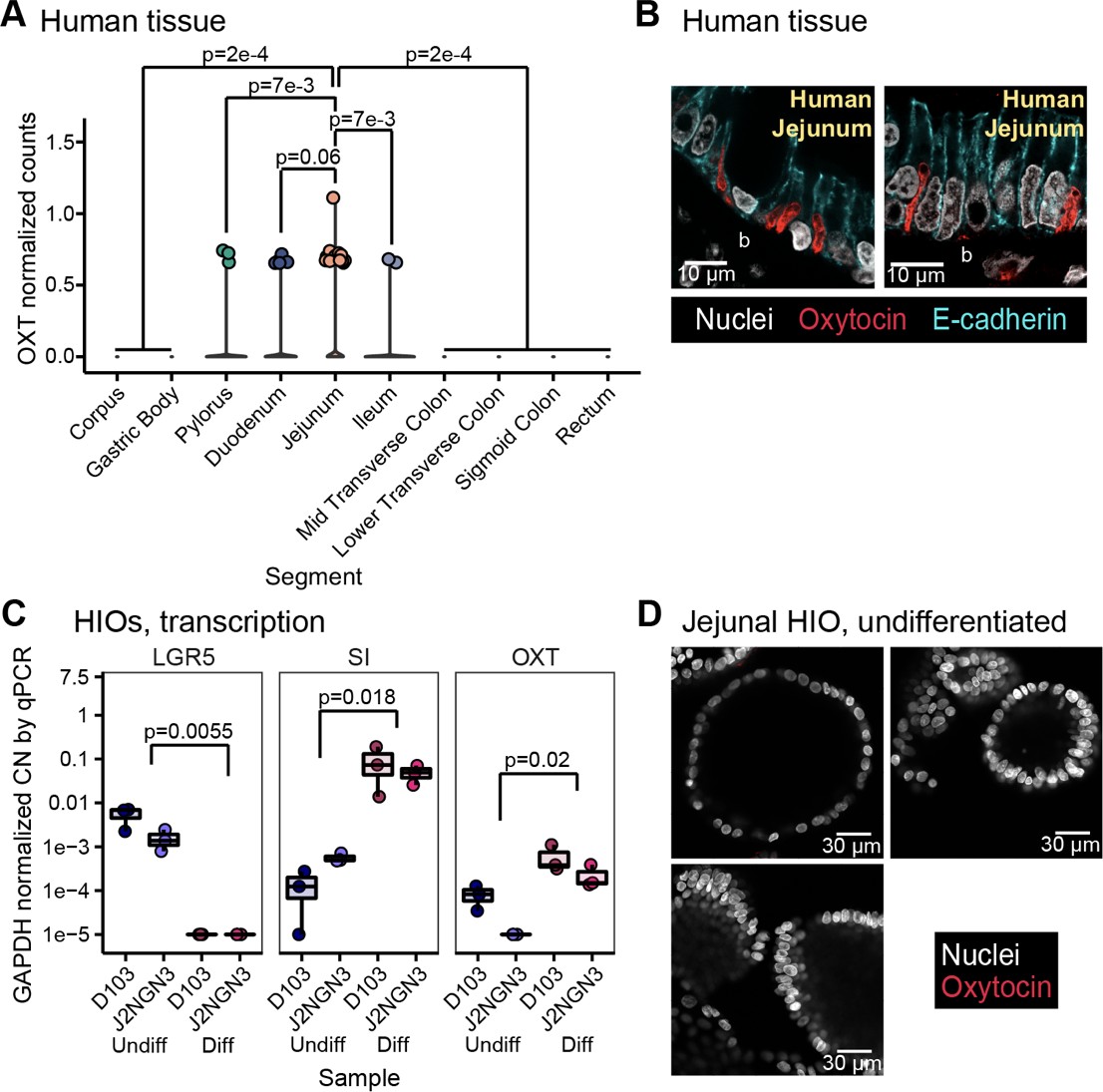


**Supplemental Figure 1: Oxytocin expression and production in the epithelium of the human gastrointestinal tract. A)** Log normalized counts of oxytocin expression in human intestinal epithelial cells reported by the scRNA-Seq data of the Human Cell Landscape^1^. Significance values reflect the number of rarefactions (of 10,000) in which the comparison had a *p* value >0.05 by a Dunn Test with a Benjamini-Hochberg correction. These *p* values were similar whether the number of cells expressing oxytocin or oxytocin expression counts were used. Only significance values <0.05 are shown. **B)** Oxytocin visualized by immunofluorescence imaging in 35 μm sectioned human jejunum. Basolateral (b) sides are labeled. **C)** Copy number (CN) of *LGR5* (stem cell marker), *SI* (differentiation marker), and *OXT* transcripts by rt-qPCR, normalized to *GAPDH* CN in undifferentiated and differentiated duodenal (D103) and jejunal (J2-*NGN3*, differentiated but not induced to increase enteroendocrine cells) organoids. Dots represent averaged triplicate qPCR data each from a separate pooled batch of three 3D organoid wells.

Where product could not be amplified during rt-qPCR, a *GAPDH* CN normalized value of 1e-5 was used. Significance values were determined from the least squares means derived from a linear mixed model with pairwise comparisons corrected using a Benjamini-Hochberg multiple testing correction (see **Supplemental Tables 2** and **3**). **D)** Oxytocin visualized by confocal immunofluorescence in undifferentiated 3D J2-*NGN3* organoids. In the images, DAPI stained nuclei are shown in white, oxytocin staining in red, and E-cadherin staining in cyan (B only). Undiff, undifferentiated. Diff, differentiated. A: all regions rarefied to 1,281 cells, each region was derived from a single patient; C: *n* = 6 HIO batches, three each from two different HIO lines, with triplicate wells per batch and line combined.

**Supplemental Movie: Oxytocin visualized by immunofluorescence in differentiated 3D J2-*NGN3* organoids.** White are DAPI stained nuclei, oxytocin in red, and E-cadherin staining in cyan. Movie constructed from z-stack confocal images. Movie is available at https://[www.biorxiv.org/content/10.1101/2023.03.09.531917v1.supplementary-material](http://www.biorxiv.org/content/10.1101/2023.03.09.531917v1.supplementary-material)


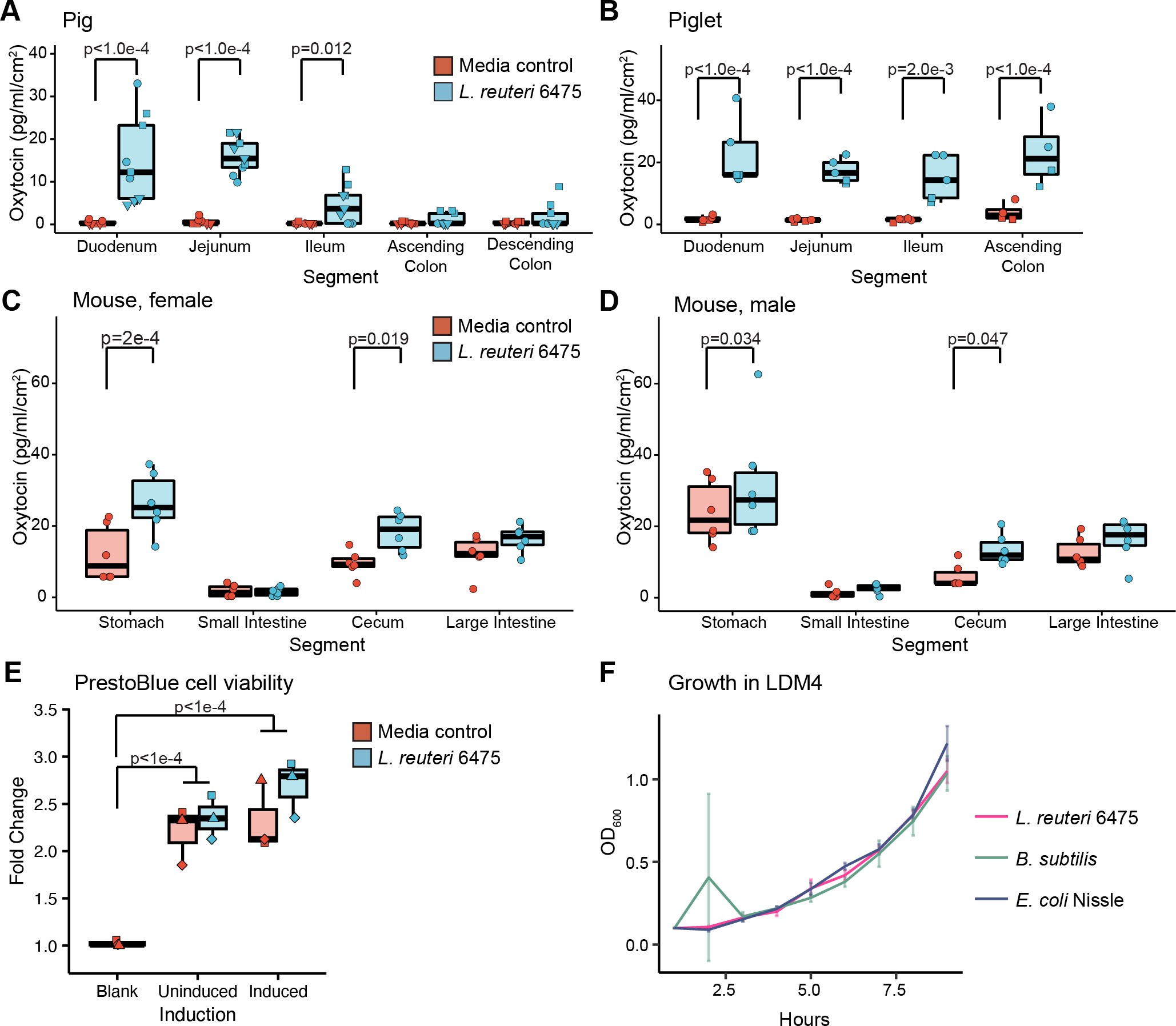


**Supplemental Figure 2: Oxytocin secreted from pig, piglet, and mouse intestinal tissue in response to *L. reuteri*-conditioned medium.** Oxytocin measured by ELISA and normalized by tissue surface area secreted from *ex vivo* **A)** pig, **B)** piglet, **C)** female mouse, and **D)** male mouse intestinal tissue. Point shapes indicate unique animals (in A and B). Each point is a unique animal in C and D. Treatment groups are colored as indicated in A and C. Mouse data with the sexes combined is also shown in Figure 2C. **E)** PrestoBlue cell viability assay of uninduced or induced *J2-NGN3* HIOs treated with LDM4 or *L. reuteri* 6475 conditioned medium. LDM4 was used directly in the PrestoBlue assay as the “Blank” and used to normalize the data. Values higher than the “blank” are indicative of metabolic activity and cell viability. **F)** Growth curves of *L. reuteri* 6475, *E. coli* Nissle, and *B. subtilis* in LDM4.

Significance values were determined from the least squares means derived from a linear mixed model with pairwise comparisons corrected using a Benjamini-Hochberg multiple testing correction (see **Supplemental Tables 2** and **3**). A: *n* = 3 animals per region and condition with three replicate tissues (9 datapoints total); B: *n* = 2 animals per region and condition with three replicate tissues (6 datapoints total); C, D: *n* = 6 animals per region and condition; E: *n* = 3 HIOs batches treated with three different preparations of the LDM4 or *L. reuteri* conditioned medium, with each point representing averaged triplicate HIO wells; F: *n* = 3 independent cultures, averaged data with the standard deviation plotted*.*


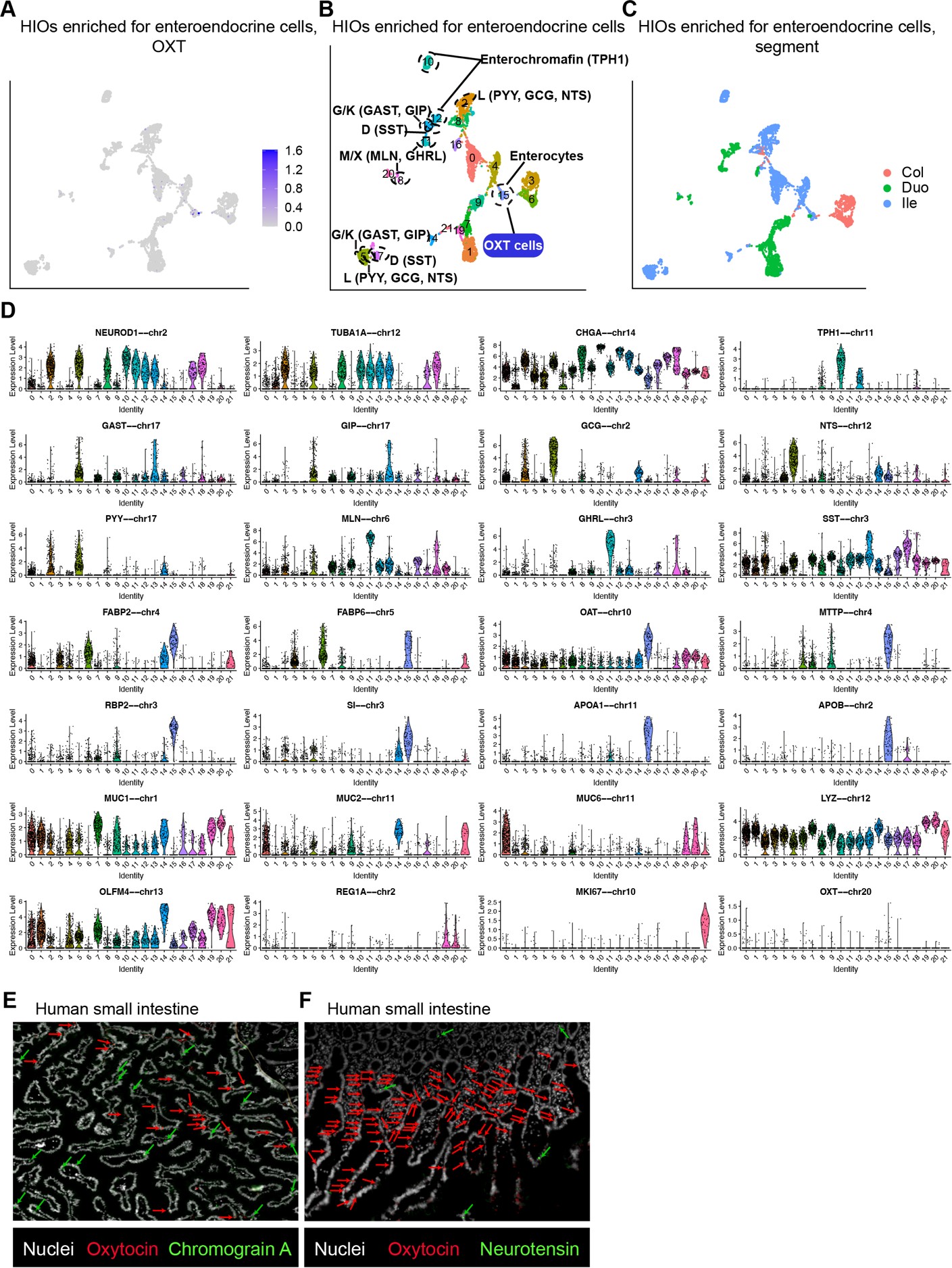


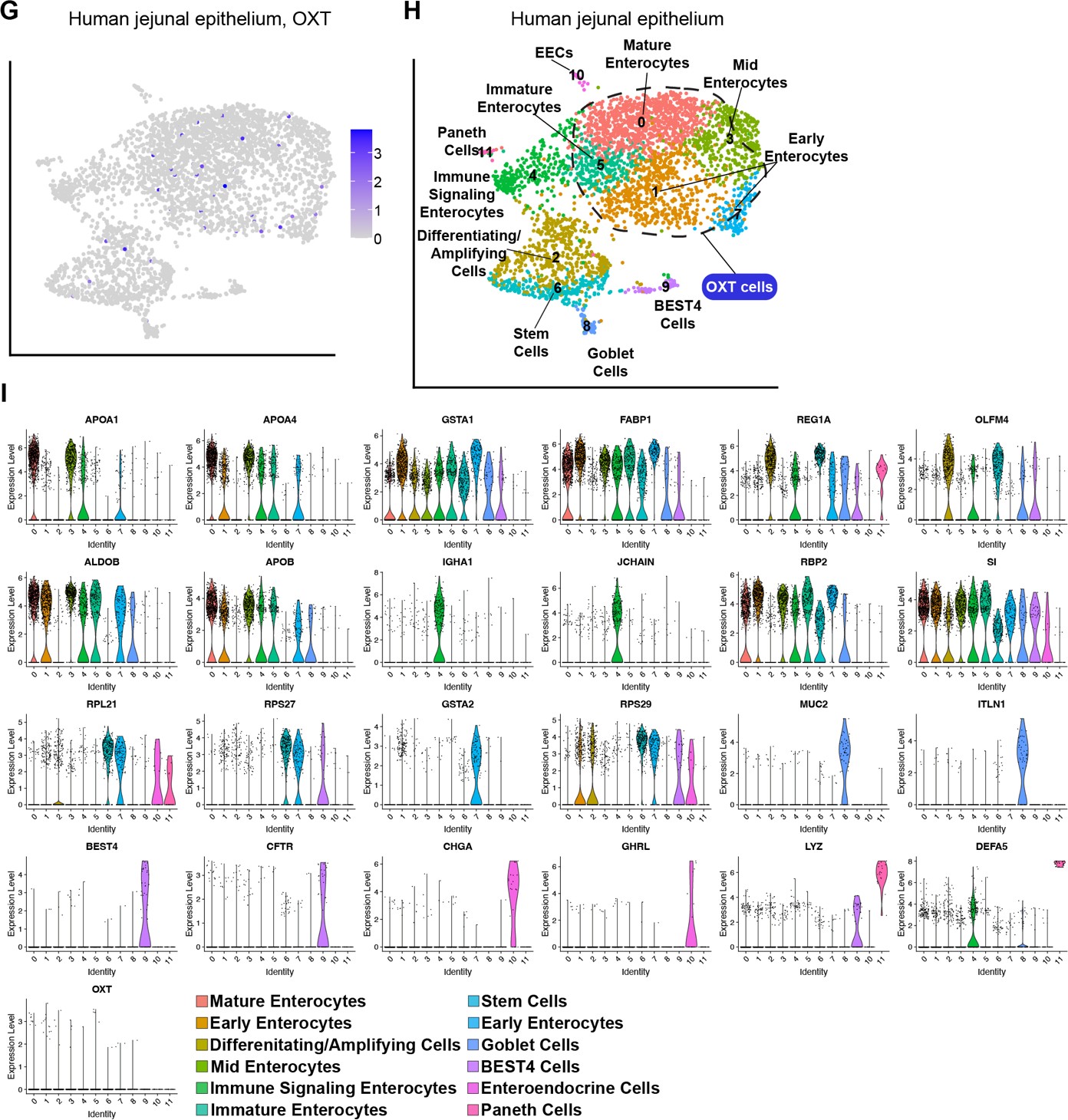


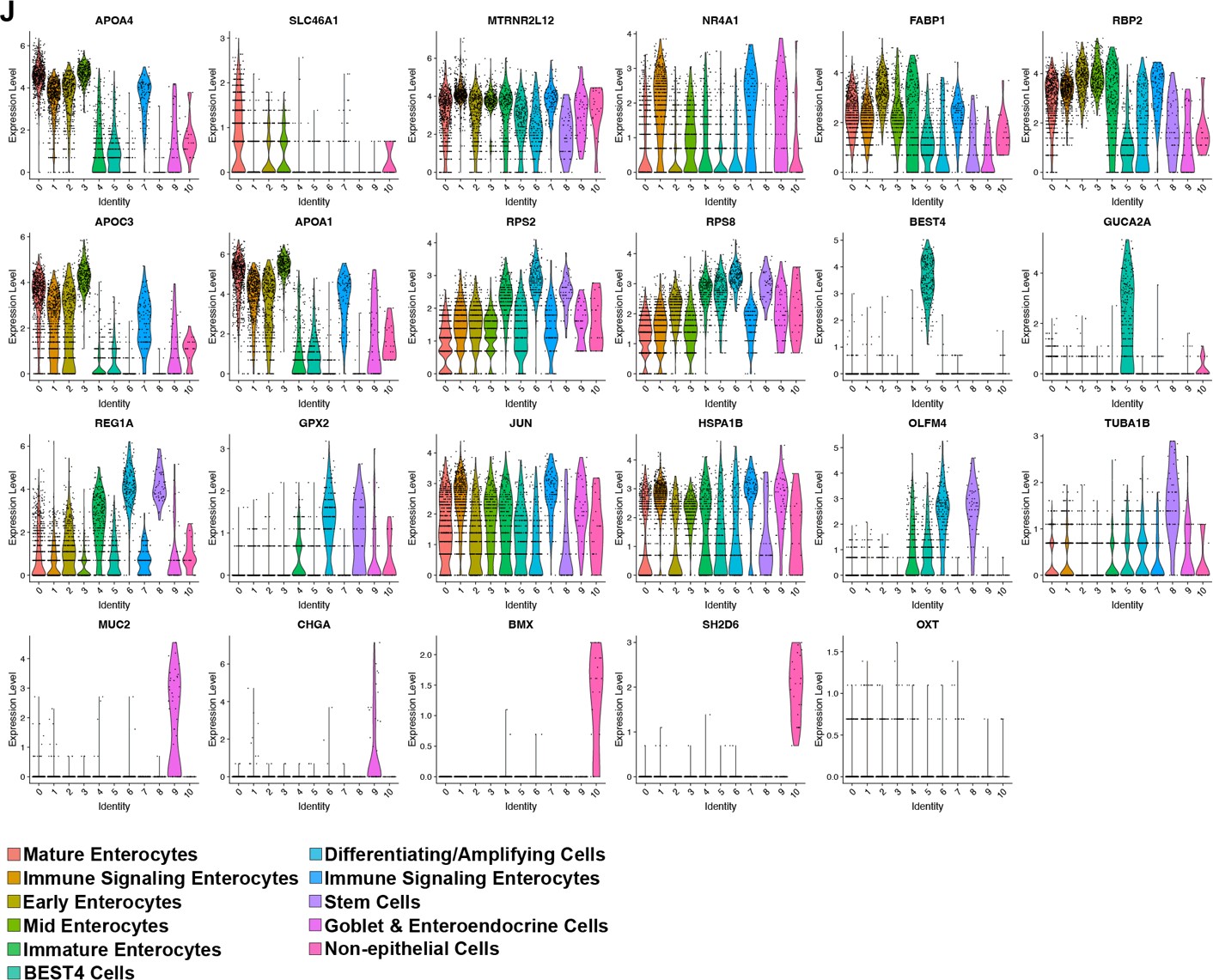


**Supplemental Figure 3: Oxytocin transcription in enterocytes**. UMAP of Beumer et al 2020^2^ scRNA- Seq data labeled with **A)** oxytocin counts, **B)** identified cell clusters, or **C)** colored by intestinal segment.

1. Gene enrichments used to annotate clusters in B. Also see **Supplemental Table 4**. Oxytocin (red) and
2. chromogranin A (green) or **F)** neurotensin (green) labeled in 6 μm sectioned human small intestinal tissue. Nuclei stained with DAPI are shown in white. UMAP of Human Cell Landscape scRNA-Seq data^1^ labeled with **G)** oxytocin counts or **H)** identified cell clusters**. I)** Gene enrichments used to annotate clusters in H. Also see **Supplemental Table 5**. **J)** Gene enrichments used to annotate clusters in **Figure 3D**. Also see **Supplemental Table 6**. A-D: *n* = 4,383 cells from 19 different combinations of cell line and treatment; G-I: *n* = 3,508 cells from a single patient; J: *n* = 2,791 cells from 4 different patients.


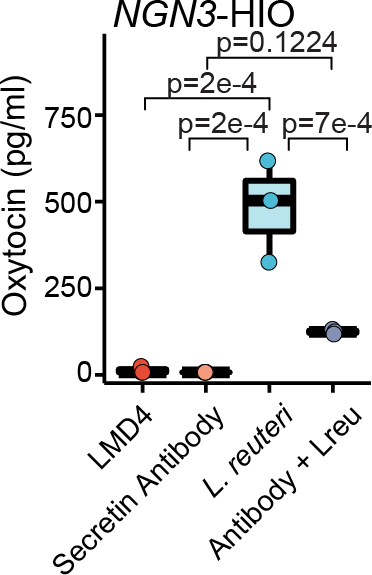


**Supplemental Figure 4: Blocking the secretin receptor eliminates *L. reuteri*-induced oxytocin release.** Oxytocin measured by an ELISA released from induced J2-*NGN3* HIOs treated with bacterial medium control (LDM4), secretin receptor antibody (1:100 dilution), and/or *L. reuteri*- conditioned medium. Significance values were determined from the least squares means derived from a linear model with pairwise comparisons corrected using a Benjamini-Hochberg multiple testing correction (see **Supplemental Tables 2** and **3**). *n* = 3 replicate monolayers per condition from one HIO batch.


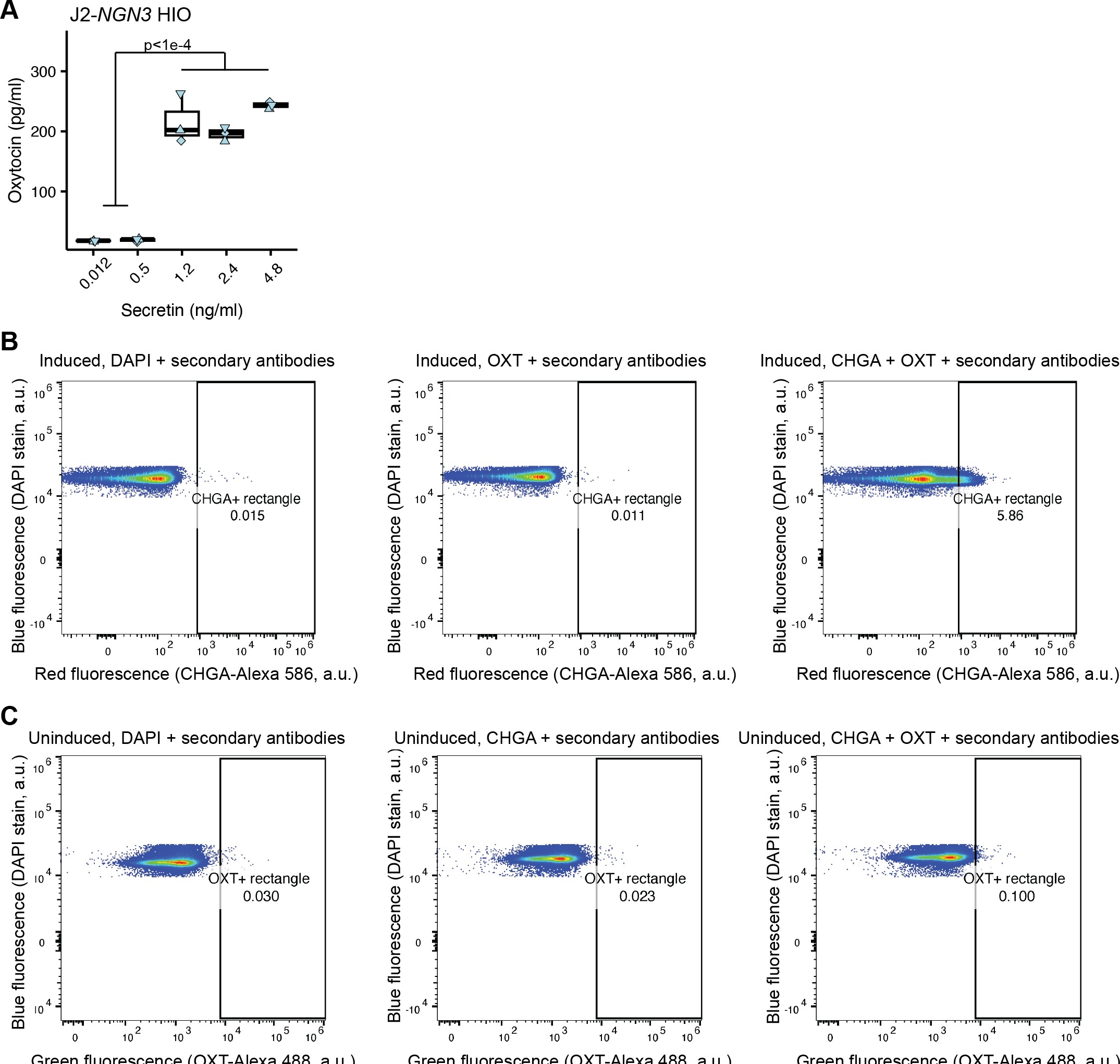


**Supplemental Figure 5: Secretin titration and gating strategy for flow cytometry. A)** Oxytocin release from induced *J2-NGN3* HIOs treated with varying concentrations of secretin. Shape reflects HIO batch. Example gating strategies for **B)** CHGA and **C)** OXT. For uninduced and induced J2-*NGN3* HIOs separately, gating for CHGA or OXT was established by looking at all staining controls not containing the CHGA or OXT primary antibody and setting the gate such that ≤ 0.05% positive cells were observed in the relevant channel. In both A and B, the left and middle plots are examples of staining controls and the rightmost plots are fully stained samples used for quantification. Cells shown are those after selecting single cells and those in G1. Significance values were determined from the least squares means derived from a linear mixed model with pairwise comparisons corrected using a Benjamini-Hochberg multiple testing correction (see **Supplemental Tables 2** and **3**). A: *n =* 3 HIO batches with triplicate monolayers averaged for each point.

**Supplemental Table 1: scRNA-Seq datasets surveyed for *OXT* expression.**

| **Dataset** | **Species** | **Age group** | **Type** | **Intestinal region(s)** | **Total OXT+**  **cells** | **Total cells** |
| --- | --- | --- | --- | --- | --- | --- |
| Grün et al. Nature  2015 | Mouse | Adult | Organoids | Small intestine | 1 | 1680 |
| Haber et al. Nature  2017 | Mouse | Adult | Epithelial tissue  and organoids | Small intestine | 11 | 51897 |
| Beumer et al Nature  Cell Biology 2018 | Mouse | Adult | Organoids | Small intestine | 6 | 3072 |
| Fujii et al Cell Stem  Cell 2018 | Human | Adult | Epithelial tissue  and organoids | Ileal | 29 | 9281 |
| Beumer et all Cell  2020 | Human | Adult | Organoids | Small & large  intestine | 125 | 8448 |
| Han et al Nature  2020 | Human | Adult | Whole tissue | Stomach, small,  & large intestine | 52* | 29700 |
| Fawkner-Corbett et  al Cell 2021 | Human | Fetal | Whole tissue | Small & large  intestine | 3082* | 63385 |
| Elmentaite et al  Nature 2021 | Human | Fetal,  adult | Whole tissue | Small & large  intestine | 8523* | 142113 |
| Li et al. Cell  Regeneration 2022 | Mouse | Adult | Epithelial tissue | Ileum | 2 | 1144 |
| Li et al. Cell  Regeneration 2022 | Rat | Adult | Epithelial tissue | Ileum | 0 | 3047 |
| Li et al. Cell  Regeneration 2022 | Pig | Adult | Epithelial tissue | Ileum | 0 | 913 |
| Li et al. Cell  Regeneration 2022 | Macaque | Adult | Epithelial tissue | Ileum | 220 | 891 |

*count post-filtering for epithelial cells

**Supplemental Table 2: Statistical models.** Provided in Excel document.

**Supplemental Table 3: Effect sizes.** Provided in Excel document.

**Supplemental Table 4: Genes differentially expressed among clusters permitting cluster annotation in the Beumer et al 2020 dataset.** Provided in Excel document.

**Supplemental Table 5: Genes differentially expressed among clusters permitting cluster annotation in the jejunum epithelium Human Cell Landscape dataset**. Provided in Excel document.

**Supplemental Table 6: Genes differentially expressed among clusters permitting cluster annotation in the jejunum Gut Cell Atlas dataset.** Provided in Excel document.

**Supplemental Table 7: Genes differentially expressed between cells expressing *OXT* and not in the jejunum Gut Cell Atlas dataset.** Provided in Excel document.

**Supplemental Table 8: Antibodies used for imaging and flow cytometry.**

| **Antibody type** | **Target** | **Host** | **Company** | **Catalog #** | **Dilution** |
| --- | --- | --- | --- | --- | --- |
| Primary | Chromogranin  A | Mouse | Santa Cruz, USA | sc-393941 | 1:100 (6 µm tissue); 1:50 (flow  cytometry) |
| Primary | Neurotensin | Mouse | Santa Cruz, USA | sc-377503 | 1:100 (6 µm tissue) |
| Primary | Oxytocin | Rabbit | Sigma-Aldrich,  USA | HPA071892 | 1:100 (3D organoids) |
| Primary | Oxytocin | Rabbit | ImmunoStar, USA | 20068 | 1:500 (6 µm tissue); 1:100 (35 µm tissue); 1:4000 (3D organoids); 1:100 (flow  cytometry) |
| Primary | Sucrase  isomaltase | Mouse | Santa Cruz, USA | sc-393470 | 1:25 (6 µm tissue) |
| Primary | MTP | Mouse | Santa Cruz, USA | sc-515742 | 1:100 (6 µm tissue) |
| Primary | OAT | Mouse | Santa Cruz, USA | sc-374243 | 1:25 (6 µm tissue) |
| Primary | APOA1 | Mouse | Santa Cruz, USA | sc-376818 | 1:25 (6 µm tissue) |
| Primary | Aldolase B | Mouse | Santa Cruz, USA | sc-393278 | 1:25 (6 µm tissue) |
| Secondary | Anti-mouse Alexa Fluor  488 | Goat | Life Technologies, USA | A-11001 | 1:300 (6 µm tissue) |
| Secondary | Anti-mouse  Alexa Fluor 568 | Goat | Invitrogen, USA | A-11004 | 1:600 (flow cytometry) |
| Secondary | Anti-rabbit  Alexa Fluor 488 | Goat | Invitrogen, USA | ab150077 | 1:800 (3D organoids and flow cytometry) |
| Secondary | Anti-rabbit  Rhodamine Red-X | Goat | Jackson  ImmunoResearch, USA | AB_2338028 | 1:200 (6 µm tissue) |
| Primary- Conjugated | Alexa Fluor 647  conjugated E-  cadherin | Mouse | BD Pharmingen, USA | 560062 | 1:50 (6 µm tissue); 1:10 (35 µm tissue) |
| Stain | NucBlue Fixed Cell  Stain | NA | Invitrogen, USA | R37606 | 0.07 to 0.1x (6 µm tissue); undiluted (35 µm tissue); 0.07x (3D organoids); 0.07x (flow  cytometry) |

**Supplemental Table 9: Primers for rt-qPCR.**

| **Target** | **Forward**  **Sequence** | **Reverse Sequence** | **Purpose** | **Citation** |
| --- | --- | --- | --- | --- |
| *OXT* | GCTGAAACTTGA  TGGCTCCG | TTCTGGGGTGGCT  ATGGG | Detect *OXT* | Wang et al. 2008. *Molecular*  *Psychiatry.* **13**: 786-799. |
| *LGR5* | CTCCCAGGTCTG  GTGTGTTG | GAGGTCTAGGTAG  GAGGTGAAG | Marker for stem cells | Chang-Graham et al. 2019.  *CMGH*. **8**: 209-229. |
| *SI* | CATCCTACCATG TCAAGAGCCAG | GCTTGTTAAGGTG GTCTGGTTTAAAT  T | Marker for enterocytes | Sclafani et al. 2007. *Proc Natl Acad Sci USA*. 104:14887-  14888. |
| *GAPDH* | ACCACAGTCCAT  GCCATCAC | TCCACCACCCTGT  TGCTGTA | qPCR normalization | Wang et al. 2015. *Cell &*  *Bioscience*. **5:**3. |
| *CHGA* | TGTAGTGCTGAA  CCCCCACC | CTCTCGCCTTTCC  GGATCT | Marker for  enteroendocrine cells | Chang-Graham et al. 2019.  *CMGH*. **8**:209-229. |

**References**

1. Han X, Zhou Z, Fei L, Sun H, Wang R, Chen Y, Chen H, Wang J, Tang H, Ge W, et al. Construction of a human cell landscape at single-cell level. Nature 2020; 581:303–9.
2. Beumer J, Puschhof J, Bauzá-Martinez J, Martínez-Silgado A, Elmentaite R, James KR, Ross A, Hendriks D, Artegiani B, Busslinger GA, et al. High-Resolution mRNA and Secretome Atlas of Human Enteroendocrine Cells. Cell 2020; 181:1291-1306.e19.
